# Changing Interpretations of Emotional Expressions in Working Memory With Aging

**DOI:** 10.1037/emo0000481

**Published:** 2018-10-15

**Authors:** Robert M. Mok, Jasper E. Hajonides van der Meulen, Emily A. Holmes, Anna Christina Nobre

**Affiliations:** 1 Department of Experimental Psychology, and Oxford Centre for Human Brain Activity, Department of Psychiatry, Wellcome Centre for Integrative Neuroimaging, University of Oxford; 2 Department for Clinical Neuroscience, Karolinska Institutet; 3 Department of Experimental Psychology, and Oxford Centre for Human Brain Activity, Department of Psychiatry, Wellcome Centre for Integrative Neuroimaging, University of Oxford

**Keywords:** emotion, working memory, aging, positivity bias, facial expressions

## Abstract

Working memory (WM) shows significant decline with age. It is interesting to note that some research has suggested age-related impairments can be reduced in tasks that involve emotion-laden stimuli. However, only a few studies have explored how WM for emotional material changes in aging. Here we developed a novel experimental task to compare and contrast how emotional material is represented in older versus younger adults. The task enabled us to separate overall WM accuracy from emotional biases in the content of affective representations in WM. We found that, in addition to overall decline in WM performance, older adults showed a systematic positivity bias in representing information in WM relative to younger adults (positivity effect). They remembered fearful faces as being less fearful than younger adults and interpreted ambiguous facial expressions more positively. The findings show that aging brings a type of positivity bias when picking up affective information for guiding future behavior.

Working memory (WM) is an essential cognitive function, enabling us to hold information in mind for goal-oriented behavior (Baddeley & Hitch, 1974). It plays a role in a wide range of cognitive processes, including attention (Desimone & Duncan, 1995) and planning (Baddeley & Hitch, 1974). WM has highly limited capacity, which shows significant declines with age (Cowan, Naveh-Benjamin, Kilb, & Saults, 2006), which, given its fundamental role across cognitive domains, can have deleterious effects on everyday life (Davis, Marra, Najafzadeh, & Liu-Ambrose, 2010). However, a growing body of research suggests that WM capacity is not fixed, and can be modulated by factors such as attention (Griffin & Nobre, 2003; Landman, Spekreijse, & Lamme, 2003). Notably, recent studies have demonstrated that older adults retain flexibility over WM, such that the ability to use attention to improve WM performance shows little age-related impairment (Gilchrist, Duarte, & Verhaeghen, 2016; Mok, Myers, Wallis, & Nobre, 2016; Newsome et al., 2015; Souza, 2016). However, these studies have used affectively neutral stimuli, which leaves open the possibility that stimulus content might also influence WM performance in older adults.

WM may also be modulated by affective content, but only a few studies have explored this in the context of aging (Bermudez & Souza, 2017; Hartley, Ravich, Stringer, & Wiley, 2015; Mammarella, Borella, Carretti, Leonardi, & Fairfield, 2013; Mikels, Larkin, Reuter-Lorenz, & Carstensen, 2005; Truong & Yang, 2014). Findings from perceptual and long-term memory tasks suggest that older adults retain sensitivity to the emotional valence of stimuli. Considerable research has shown that negatively valenced stimuli can capture attention and boost perceptual performance in younger adults (emotional salience effect; e.g., Öhman, Flykt, & Esteves, 2001; Phelps, Ling, & Carrasco, 2006), and this boost seems to be retained in older adults (Fung & Carstensen, 2003; Mather & Knight, 2006; Murphy & Isaacowitz, 2008; Rösler et al., 2005). Both younger and older adults show better long-term memory for emotional than for neutral stimuli (e.g., Kensinger, Brierley, Medford, Growdon, & Corkin, 2002). Whereas younger adults put more weight on negative aspects of the environment, older adults have a tendency to attend to positive information (positivity effect; Mather & Carstensen, 2005; Reed, Chan, & Mikels, 2014). Older adults show superior performance on perceptual and memory tasks that use positive compared with neutral or negative stimuli (e.g., Charles, Mather, & Carstensen, 2003; Ebner & Johnson, 2009; Kellough & Knight, 2012; Reed et al., 2014).

Less is understood about the influence of stimulus valence on WM in older adults. Several studies found a benefit in WM performance for negative compared with neutral stimuli in younger adults (e.g., Jackson, Wu, Linden, & Raymond, 2009). A few studies have tested the interaction between affective content in WM and aging (Bermudez & Souza, 2017; Hartley et al., 2015; Mammarella et al., 2013; Mikels et al., 2005; Truong & Yang, 2014). These studies are limited to paradigms that use reaction-time (RT) measures, which are not ideal for testing older populations, who may have motor problems, or accuracy measures, which cannot tease apart critical questions: namely, whether age-related changes are a result of reductions in WM capacity (independent of emotional content) or are a result of how emotional information-representation changes in WM (as more or less positive or negative). For example, higher accuracy for positive versus negative stimuli might reflect better memory for positive stimuli, a tendency to see positive things as more positive, or a tendency to see negative stimuli as less negative.

In this study, we developed a new way to measure the quality of WM representations for emotional material and to assess systematic affective biases in perceiving and interpreting emotional material, for a more sensitive test of the positivity effect in aging. The task borrows from WM precision tasks, which test WM for items with features that vary continuously along a given dimension (e.g., bars with orientation of 1–180°), such that participants recall the feature (orientation) stored in memory (Bays & Husain, 2008; Zhang & Luck, 2008). The task produces sensitive estimates of the quantity and quality of items in WM (Zokaei, Burnett Heyes, Gorgoraptis, Budhdeo, & Husain, 2015). It is also possible to identify systematic biases in the patterns of responses (e.g., a bias to report clockwise or counterclockwise). We used facial expressions morphed from neutral to fearful and neutral to happy to test age-related changes in WM for emotional material. Facial expressions were chosen to produce a set of stimuli that varied on a continuous scale of positive and negative emotion. Happy faces and fearful faces were selected after consideration of their common use in previous studies on affective attentional biases (e.g., Fox, 2002; Pourtois, Grandjean, Sander, & Vuilleumier, 2004) and studies that found a relationship between attentional biases for fear-related stimuli (including faces) in anxiety (Yiend, 2010).

In the current emotional WM task, participants encoded a face into WM with an emotional expression (fearful or happy) with a certain emotional intensity. After a delay, participants used a mouse to adjust a facial expression to match the emotion type and intensity in memory. In a separate perceptual emotion-matching experiment, participants adjusted one face to match the expression of another face on the screen. Using these tasks, we compared performance accuracy and emotional bias between groups of older and younger participants to test how WM and perception for emotional material change with age. Given previous work, we might expect preserved facilitation in tasks with emotional stimuli, or only for positive stimuli, to generalize to WM and therefore mitigate against age-related deficits in WM performance in older adults. Furthermore, we might expect to measure a systematic shift in reporting the valence and emotional intensity of emotional expressions in WM, whereby fearful faces would be reported as less fearful and/or happy faces as more positive.

## Method

### Participants

Fifty-four young participants and 54 older participants volunteered to participate in the study and received compensation and travel expenses where required. The study was approved by the Central University Research Ethics Committee of the University of Oxford, and was carried out in accordance with the provisions of the World Medical Association Declaration of Helsinki (World Medical Association Declaration of Helsinki, 2013). Before taking part in the study, individuals were sent an electronic screening questionnaire, which included Spielberger’s (1983) trait-anxiety questionnaire, the State–Trait Anxiety Inventory–Trait version; STAI) and a series of questions. People who reported current use of psychoactive medication, history of recreational drug use, history of neurological illness, or took part in studies involving WM training or emotional face stimuli in the past 6 months were not invited to participate. Data from one elderly participant were excluded because of a low score (less than 26) on the Montreal Cognitive Assessment (MoCA; Nasreddine et al., 2005), and data from two other elderly participants were not saved because of a technical error. After excluding these participants, 54 younger adults (39 women, *M*_age_ = 23.42, *SEM* = .60, age range = 18–35 years) and 51 older adults (29 women, *M*_age_ = 69.25 ± .78, age range = 61–82 years) were included in the current study. All remaining participants were fluent in English, had normal or corrected-to-normal vision and hearing, and all older participants scored >26 on the MoCA, *M* = 28.16, *SEM* = .16; younger adults did not complete the MoCA. Sample sizes were determined with the aim of comparing performance between age groups and investigating the relationship between anxiety and behavioral measures. A survey of studies testing age differences in WM for emotional material that presented sufficient information for a power analysis (Mammarella et al., 2013; Truong & Yang, 2014) revealed that a minimum of 11 to 30 participants per group are required for 80% power, and a minimum of 14 to 41 participants per group are required for 90% power. A survey of previous studies that reported a relationship between measures of anxiety and attentional bias (using Pearson’s correlations) shows that the average correlation coefficient was 0.315 (Bradley, Mogg, & Millar, 2000; Fox, Cahill, & Zougkou, 2010; Fox, Mathews, Calder, & Yiend, 2007). A power calculation indicates that 77 participants provide 80% power to find a significant effect (Hulley, Cummings, Browner, Grady, & Newman, 2013). Our sample of 105 provides 90% power.

### Stimuli and Apparatus

Stimuli were adapted from faces in the NimStim Stimulus Set (http://www.macbrain.org/resources.htm) with permission. Forty-eight face stimuli (three emotional expressions for each of 16 identities) were selected. Happy, fearful, and neutral face images were cropped with an elliptical mask and morphed from neutral to fearful and from neutral to happy in 1% steps to produce faces with graded intensities of emotional expressions from 0% to 100% (see Figures S1 and S2 for examples). Ten identities were selected for the main experiment and six for the practice session. Scrambled masks were produced for each stimulus by randomly shuffling pixels within the elliptical mask (see online supplementary materials for details).

### Task Design and Procedure

#### Emotion WM task

**Figure 1 fig1:**
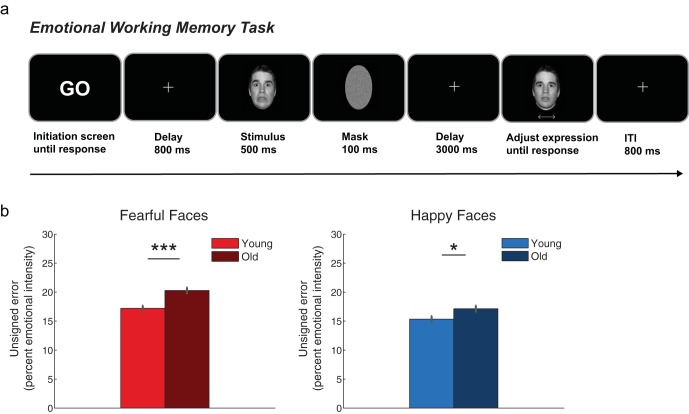
WM task schematic and WM error results. In the WM task (a), participants encoded a facial expression into WM, and maintained it over a delay of 3,000 ms. A test face with the same facial identity but a neutral facial expression (0% intensity) appeared, and participants changed the face to match the expression intensity in memory using a trackball mouse. Target faces were fearful or happy faces from 0% to 100% in emotional intensity. Emotion type was intermixed within blocks. Bar plots in (b) show WM error for fearful (red, left) and happy faces (blue, right) in the young and old participant groups. Error bars represent *SEM*. *** *p* < .001, * *p* < .05. Faces presented are part of the NimStim stimulus set, for which use for publication is permitted.

On each trial, participants encoded a face into memory and were asked to recall this face at the end of the trial. Stimuli were faces with pseudorandomly selected levels of emotional intensity values of 0% to 45% and 55% to 100% in 5% steps (leaving out 50%), with one set of intensity values for each emotion type, happy and fearful. Emotion Type conditions were intermixed within each block (see Figure 1a).[Fig-anchor fig1]

On each trial, a “GO” screen signaled to start the trial with a left mouse click. A fixation cross was presented at the center of the screen (800 ms), after which a face (500 ms) and a scrambled mask (100 ms) were presented. After a delay of 3,000 ms, a test face was presented with a neutral expression (0% intensity). Participants adjusted its expression to match the emotion type and intensity of the face in memory. Participants adjusted the face with a trackball mouse, scrolling left for one emotion and right for the other emotion (happy/fearful, counterbalanced across participants) and clicked to confirm their responses. After each block, feedback was given (percent correct; computed by 100 minus the average deviation of responses from the target emotional intensity, or mean error). Participants were asked to fixate centrally, and, if they consistently broke fixation, they were reminded to refrain from doing so at the next break. Accuracy was stressed over RT. Maximum response time was 11 s, but participants were encouraged to respond within 6 s in the interest of time and to reduce memory degradation. At 11 s, the emotional intensity on the screen was saved as the response.

Each participant completed eight blocks of 20 trials. For each emotion type (fear, happy), each emotional intensity level was presented four times, giving 80 trials per emotion type. For each participant, facial identities were pseudorandomly allocated over each emotional intensity condition and all 10 identities were included in both Emotion Type conditions. For each identity, there were 16 trials for each emotional intensity condition (from 0% to 100% with 5% steps, excluding 50%). Because there were 19 intensities per emotion type, plus a neutral face condition (0% intensity), not all intensity conditions were presented for each identity (the smallest range was 5% to 80%, but most identities spanned 0% to 100% for both emotion types). The number of emotional intensity conditions was kept constant (80 per condition per emotion).

#### Emotion-matching task

Participants were presented with a target face on the left of the screen and adjusted the face on the right to match the emotion type and intensity of the target face. As in the WM task, stimuli were happy or fearful faces with the same range of emotion-intensity conditions and identities (but the pairing of emotion-intensity conditions and identities was different). Emotion Type conditions were intermixed within each block (see Figure S3a).

Each trial began with a “GO” screen and the trial started with a mouse click. A fixation cross was presented (800 ms), after which two faces with the same identity appeared on the left and right side of the screen. Participants adjusted the expression of the face on the right to match the emotion type and intensity of the face on the left. The right face had a neutral expression, and participants adjusted the expression using a trackball mouse. As in the WM task, feedback was given after each block, accuracy stressed over RT, with the same time constraints. Eye movements were not constrained.

Each participant completed two blocks of 20 trials. For each emotion, each emotion-intensity condition was presented twice, with 20 trials per emotion type. As with the WM task, the facial identities were randomly allocated over each emotional intensity level. The identities associated with each emotion-intensity condition were different than those in the WM task.

#### Mood questionnaires

Participants completed five self-report questionnaires measuring state and trait anxiety (STAI; Spielberger, 1983), Beck’s Depression Inventory (BDI; Beck, Ward, Mendelson, Mock, & Erbaugh, 1961), and positive and negative affective states and traits (short version of the Positive and Negative Affect Scale; PANAS; Watson, Clark, & Tellegen, 1988) immediately before the experimental session.

### Data Analysis

The aim of the analyses was to characterize age-related differences in WM for emotional material in terms of error (deviation of responses from target emotional intensities), emotional bias (representing information as more positive or negative), and valence (categorical judgment of a fearful or happy face).

In both the emotion-WM and emotion-matching tasks, the target facial expressions included 0% intensity (neutral) and ranged from 1% to 100% in 5% steps (excluding 50%) in emotional intensity of the target emotion type, and participants could report emotional intensities, which ranged from 1% to 100% of the target emotion (e.g., fear). They could also report the other emotion (e.g., happy), which was recorded as a response (from −1 to −100%) or a neutral expression (0%). To calculate error, participant responses (positive or negative) were subtracted from the target emotional intensities (positive) on each respective trial, giving an error distribution—the deviation of intensities reported by participants (responses) from the actual intensity values (targets). Responses to the other emotion type produced values with a negative sign. For instance, if a target face was 50% happy and a response was 60% happy, the error was |50 – 60| = 10. A response of 40% happy would also yield an absolute error of 10. If a target was 20% fearful and the response was 15% fearful, the error would be |20 – 15| = 5. If the response was 15% happy, then the error would be |20 – (−15)| = 35. The highest possible error would be 200 (e.g., if target face was 100% fearful and the response was 100% happy), but the maximum error decreases proportionally to the valence of the stimuli (e.g., if target face was 50% fearful, the maximum error would be 150). Error was computed by taking the mean of the absolute (positive) error values across trials. Statistical tests were also performed after excluding trials in which participants reported the incorrect emotion type and trials with neutral targets. See the online supplementary materials (Trial Numbers) for details of excluded trials. Trials in which participants used up the maximum time for a response (11 s) did not have an effect on the results (for details, see online supplementary materials, Maximum Response Time Trials).

**Figure 2 fig2:**
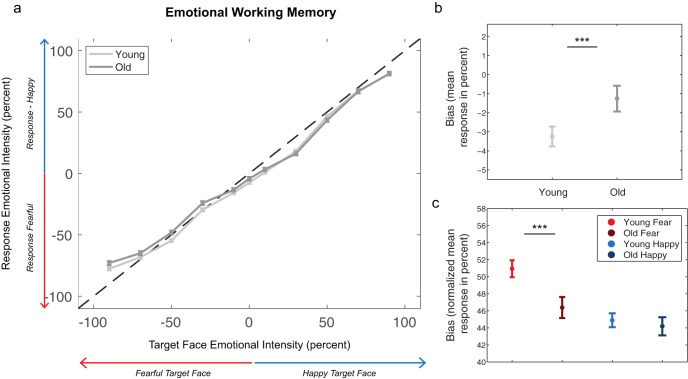
Emotional bias in WM. Responses are plotted as a function of the target-face emotion type and emotional intensity in (a), with negative values representing intensity values of fearful faces and positive values representing intensity values of happy faces. Responses were binned into five equal bins for fearful faces (from −100 to −20% in 20% steps, with the 20% bin including −20 to −1%) and five bins for happy faces (from 20% to 100% in 20% steps) and a 0% bin with only neutral faces for visualization. Perfect performance corresponds to responses on the diagonal (dotted line). On the right side of zero (*y* axis), responses above the line mean that faces were reported to be happier than happy-face targets, whereas responses below the line mean that faces were reported to be less happy than targets. On the left side of zero, responses below the line mean that faces were reported to be more fearful than fearful-face targets, whereas responses above the line mean that faces were reported as less than fearful-face targets. The bias is shown in (b), computed by taking the mean of each participant’s raw psychometric curve—note that (a) is binned for visualization. Bias for each of the emotion types is plotted in (c). Responses for fearful faces were flipped to have a positive sign, and trials with neutral faces were excluded. Mean response was computed for each emotion type (from 1% to 100%) and normalized by subtracting 50 (see Data Analysis for details). *** *p* < .001.

Emotional bias was derived from the shift in the psychometric function of responses. Participants’ responses were plotted as a function of the actual emotion type and emotional intensity of the target face, with negative values representing intensities of fearful faces and positive values representing intensities of happy faces (see Figure 2a). Note that “response” values are the actual emotional intensity values that participants reported, unlike the error values above, which were calculated relative to the target. To obtain an overall measure of bias, we computed the mean of this curve (mean response across all intensity conditions, −100% to 100%). Positive bias values corresponded to the tendency to report faces as either more positive or less negative (or both); negative bias values reflected the tendency to report faces as less positive or more negative (or both), and a value of zero corresponded to no bias. For instance, if a target face was 50% happy and a response was 60% happy or 40% happy, the bias value on those trials would be 10% and −10% respectively. If a target face was 20% fearful and a response was 15% happy, the bias would be 35% (15 – [−20]), whereas if the target face were 15% happy and response was 20% fearful, the bias would be −35% (−20 – 15). Note that intensity values of happy faces are positive, and values of fearful faces are negative. The most negative possible bias would be −200% (if target face was 100% happy and the response was 100% fearful) and most positive bias would be 200% (if target face was 100% fearful and the response was 100% happy), but would normally be lower. Statistical tests were also performed after excluding trials in which participants reported the incorrect emotion type, because trials in which participants judged happy faces to be fearful might contribute to an overall negative bias, and trials in which participants judged fearful faces to be happy might contribute to an overall positive bias.[Fig-anchor fig2]

To test for biases that stemmed specifically from the fearful or happy conditions, bias was computed for emotion types separately. First, responses for the fearful faces were flipped to positive to be compared with happy bias values. Second, the trials with neutral faces (0% intensity) were excluded. The mean response was computed across emotional intensities for each emotion type (from 1% to 100%), then normalized by subtracting 50 to match the overall bias measure, so that a value of zero would reflect no bias.

To characterize judgments of valence (categorical judgment of fearful or happy), we separated responses into the correct and incorrect emotion types, which we excluded in a subset of the analyses above. Reporting the incorrect emotion occurred when a participant adjusted a face to the wrong emotion type (e.g., reported 25% fearful face but the target was a happy face). To inspect the effect of emotional intensity on valence judgments, trials were binned into five equal bins of emotional intensity (i.e., 1–20, 21–40, 41–60, 61–80, 81–100). Proportion correct was computed for each target emotion intensity bin (e.g., a proportion of .7 correct for a given intensity bin meant participants reported the correct emotion type 70% of the time and the incorrect emotion type 30% of the time).

A mixed repeated-measures analysis of covariance (ANCOVA) was conducted on WM error, with within-subject factor Emotion Type (fearful, happy), between-subjects factor Age (young, old), and continuous factor Anxiety (STAI trait). Anxiety was included to test for the relationship between behavior and mood. A mixed repeated-measures ANCOVA was performed on emotional bias to test between-subjects factor Age with continuous factor Anxiety. To test whether bias effects were driven by happy or fearful faces, a mixed repeated-measures ANCOVA was conducted on WM bias for happy and fearful face conditions with Emotion Type, Age, and Anxiety factors. For the emotion-matching task, the same ANCOVAs listed above were conducted. All ANCOVAs above were recomputed after excluding trials to the incorrect emotion type and neutral target face trials. A mixed repeated-measures ANCOVA was conducted on proportion of correctly categorized faces in the emotion WM and emotion-matching task separately, with Emotion Type, Intensity (1–20, 21–40, 41–60, 61–80, 81–100), Age, and Anxiety factors. Gender was included in all ANCOVAs as a covariate of no interest. Degrees of freedom were corrected using Greenhouse–Geisser estimates of sphericity when normality assumptions were violated.

Paired *t* tests were used to test for paired-condition differences, and independent-samples *t* tests to compare between age groups. To test for the direction of linear contrasts, we used a one-sample *t* test to see if slopes and differences in slopes (between emotion types) were different from zero. Cohen’s *d* was used to determine effect sizes. All analyses conducted are reported in this section. Confidence intervals for Cohen’s *d* and η_p_^2^ (for ANCOVAs) were calculated using the MBESS package in R (for between-subjects effects), or from custom R code (for within-subject effects, see https://github.com/Lakens/perfect-t-test).

Statistical analyses were conducted in Matlab R2015a, Matlab’s Statistics Toolbox, and R Version 3.2.1 (R Core Team, Vienna, Austria) using the afex package (Singmann, Bolker, & Westfall, 2015) and MBESS package. The code to run the experiment (Matlab, Psychtoolbox), data-analysis code (Matlab, R), and the behavioral data are available online (see https://osf.io/a47xe/). The authors are happy to share the data and the experimental scripts. However, before we are able to share the stimuli, which are necessary for the task, permission needs to be obtained to use the NimStim faces from the original creators. These stimuli are for research purposes only (see http://danlab7.wixsite.com/nimstim)^1^.

## Results

### Accuracy for Matching Emotional Faces in WM and Perception

Participants completed the emotion-WM task with high accuracy (percent error: *M*_youngfear_ = 17.20, *SEM* = .48, *M*_oldfear_ = 20.30, *SEM* = .53; *M*_younghappy_ = 15.34, *SEM* = .53, *M*_oldhappy_ = 17.13, *SEM* = .56) and showed better WM performance for happy than fearful faces, Emotion Type: *F*(1, 101) = 40.20, *p* < .001, η_p_^2^ = .28, 90% CI [.17, .39]. Older adults showed a general deficit in WM for emotional content, Age: *F*(1, 101) = 14.10, *p* < .001, η_p_^2^ = .12, 90% CI [.04, .22], which was more prominent for fearful faces, Age × Emotion Type interaction: *F*(1, 101) = 4.84, *p* = .03, η_p_^2^ = .05, CI 90% [.002, .12]; young versus old fear: *t*(101.04) = −4.29, *p* < .001, *d* = −.84, 95% CI [−1.24, −.44]; happy: *t*(102.43) = −2.31, *p* = .023, *d* = −.45, 95% CI [−0.84, −0.06]; see Figure 1b. The results were similar after excluding trials in which participants reported the incorrect emotion type (error: *M*_youngfear_ = 16.42, *SEM* = .48, *M*_oldfear_ = 18.54, *SEM* = .46; *M*_younghappy_ = 11.97, *SEM* = .33, *M*_oldhappy_ = 13.58, *SEM* = .40), with better performance for happy than for fearful faces, Emotion Type: *F*(1, 101) = 221.61, *p* < .001, η_p_^2^ = .69, 90% CI [.60, .74]. Older adults were still significantly worse than the younger group, Age: *F*(1, 101) = 14.35, *p* < .001, η_p_^2^ = .12, 90% CI [.04, .22], but there was no longer an interaction between Age and Emotion Type, *F*(1, 101) = .89, *p* = .35, η_p_^2^ = .01, 90% CI [0, .06].

Although there were fewer trials in the perceptual matching task, the pattern of results was similar to the WM task (error: *M*_youngfear_ = 10.42, *SEM* = .42, *M*_oldfear_ = 12.67, *SEM* = .52; *M*_younghappy_ = 7.09, *SEM* = .34, *M*_oldhappy_ = 8.15, *SEM* = .40), with better performance for happy than for fearful matching, Emotion Type: *F*(1, 101) = 125.34, *p* < .001, η_p_^2^ = .55, 90% CI [.44, .63]. Older adults were worse than younger adults at matching the emotional faces, Age: *F*(1, 101) = 10.00, *p* = .002, η_p_^2^ = .09, 90% CI [.02, .18]. After excluding trials in which participants erroneously reported the incorrect emotion type, the pattern of performance was similar, error: *M*_youngfear_ = 10.23, *SEM* = .43, *M*_oldfear_ = 12.06, *SEM* = .49; *M*_younghappy_ = 6.72, *SEM* = .32, *M*_oldhappy_ = 7.54, *SEM* = .33; Emotion Type: *F*(1, 101) = 145.48, *p* < .001, η_p_^2^ = .59, 90% CI [.49, .66], with a strong effect of Age, *F*(1, 101) = 7.40, *p* = .008, η_p_^2^ = .07, 90% CI [.01, .15].

### Age-Related Shifts of Emotional Bias in WM

Younger adults exhibited a stronger negative shift in their WM psychometric curves than older adults, *M*_young_ = −3.25, *SEM* = .52; *M*_old_ = −1.26 *SEM* = .68; *F*(101) = 6.55, *p* = .01, η_p_^2^ = .06, 90% CI [.007, .15]; see Figure 2a–b. They reported fearful faces as more emotionally intense than older adults: see Figure 2c; Age × Emotion Type interaction, *F*(1, 101) = 6.27, *p* = .01, η_p_^2^ = .06, 90% CI [.006, .14]; young versus old: fear, *t*(97.51) = 2.87, *p* = .005, *d* = .56, 95% CI [.17, .95]; happy, *t*(94.65) = .52, *p* = .60, *d* = .10, 95% CI [–.28, 0.48]). After excluding responses to the incorrect emotion type, there was still a difference between age groups, *M*_young_ = −2.24, *SEM* = .48; *M*_old_ = –.46, *SEM* = .61; *F*(1, 101) = 5.83, *p* = .02, η_p_^2^ = .05, 90% CI [.005, .14], and the effect was likely due to the difference in fearful faces, Age × Emotion Type interaction: *F*(1, 101) = 3.20, *p* = .08, η_p_^2^ = .03, 90% CI [0, 10]; young versus old: fear, *t*(100.5) = 1.84, *p* = .069, *d* = .36, 95% CI [–.27, .74]; younger versus old: happy, *t*(88.49) = .26, *p* = .79, *d* = .05, 95% CI [–.33, .43]. There were no significant results in the perceptual matching task for these effects (see Figure S4).

### Age-Related Changes in Emotion Interpretation in WM

**Figure 3 fig3:**
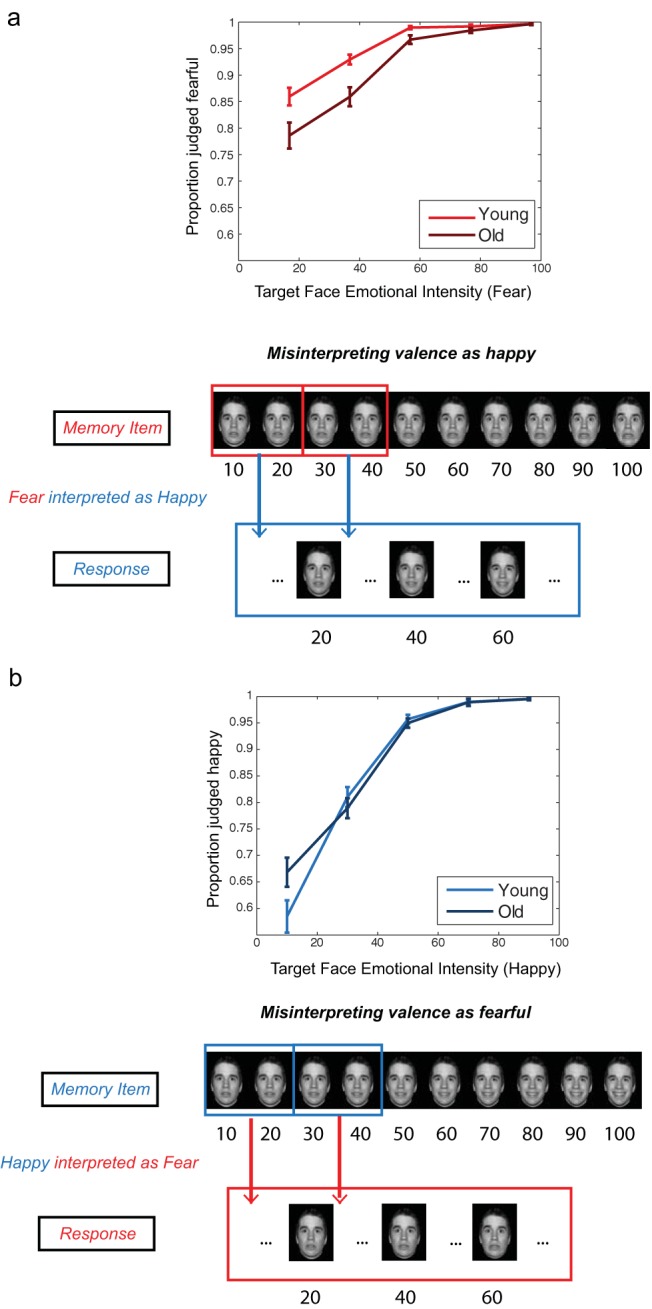
Older adults interpreted fearful faces with low emotional intensities as happy more than younger adults. Proportion of trials correctly judged as fearful in the WM task is plotted for each emotional intensity bin from 1% to 80% in 20% steps for younger and older participants in the top panel in (a). An illustration showing how low-to-medium fearful faces are sometimes judged as happy faces in the bottom panel of (a). Proportion of trials correctly judged as happy in the WM task are plotted for each emotional intensity bin in the top panel of (b) for younger and older participants, with an illustration in the bottom panel showing how low-to-medium happy faces are sometimes judged as fearful faces. Faces presented are part of the NimStim stimulus set, for which use for publication is permitted.

Although participants generally reported the correct emotion type in the emotion WM task, they also mistakenly interpreted the face to have the incorrect emotion type on a sizable proportion of trials (proportion of happy faces reported fearful: *M*_young_ = .14, *SEM* = .01/*M*_old_ = .12 ± .01; proportion of fearful faces reported happy: *M*_young_ = .05, *SEM* = .01, *M*_old_= .08, *SEM* = .01) and on a minority of the trials in the perceptual expression-matching task (proportion of happy faces reported fearful: *M*_young_ = .04, *SEM* = .01, *M*_old_ = .05, *SEM* = .01, proportion of fearful faces reported happy: *M*_young_= .04, *SEM* = .01, *M*_old_ = .07, *SEM* = .01). Figure 3 shows the proportion of trials in which participants reported the correct emotion type for each intensity bin (see Figure S6 for scatterplots that illustrate the pattern of responses across intensities).[Fig-anchor fig3]

Participants were more likely to report the correct emotion type for fearful-face trials compared with happy-face trials, Emotion Type: *F*(1, 101) = 53.78, *p* < .001, η_p_^2^ = .35, 90% CI [.22, .45]. Ambiguous, low-intensity emotional faces were more likely to be misinterpreted as the other emotion type, *F*(1.89, 191.08) = 374.25, *p* < .001; η_p_^2^ = .79, 90% CI [.74, .82]; *M*_slope_ = .07, *SEM* = .003; *t*(104) = 23.85, *p* < .001, *d* = 2.33, 95% CI [1.96, 2.70], and this effect was stronger for happy compared with fearful faces, Emotion Type × Intensity: *F*(1.74, 175.95) = 43.57, *p* < .001, η_p_^2^ = .30, 90% CI [.21, .38]; happy *M*_slope_ = .09 ± .005, *t*(104) = 18.8, *p* < .001, *d* = 1.84, 95% CI [1.52, 2.15]; fear *M*_slope_ = .04, *SEM* = .004, *t*(104) = 11.7, *p* < .001, *d* = 1.15, 95% CI [.90, 1.39]; *M*_slopediff_ =.049, *SEM* = .007, *t*(104) = −7.42, *p* < .001, *d* = 1.09, 95% CI [.77, 1.42].

Crucially, older adults were more likely to judge a fearful face as a happy one in the WM task, Age × Emotion Type interaction: *F*(1, 101) = 10.06, *p* = .002, η_p_^2^ = .09, 90% CI [.02, .18]; *t*(77.75) = −3.56, *p* < .001, *d* = −.70, 95% CI [−1.09, −.30], whereas both age groups judged happy faces as fearful to a similar extent, *t*(102.98) = .80, *p* = .43, *d* = .15, 95% CI [–.23, .54]. This effect was modulated by the emotional intensity of the face stored in WM, Age × Emotion Type × Intensity interaction: *F*(1.74,175.95) = 8.17, *p* < .001, η_p_^2^ = .07, 90% CI [.02, .14], whereas older adults tended to judge fearful faces with low-to-medium intensities as happier than did the younger adults, *M*_slopediff_ = .02, *SEM* = .04; *t*(82.15) = 2.87, *p* = .005, *d* = .56, 95% CI [0.17, 0.95], but not for the happy faces, *M*_slopediff_ = −.01, *SEM* = .04; *t*(102.18) = −1.50, *p* = .14, *d* = −.29, 95% CI [–.68, .09].

In the perceptual matching task, participants showed the opposite pattern for Emotion Type, such that they mistakenly interpreted fearful faces as happy more than they judged happy faces as fearful, Emotion Type: *F*(1, 101) = 8.71, *p* = .004, η_p_^2^ = .08, 90% CI [.02, .17]. Participants incorrectly reported the Emotion Type for faces with low emotional intensity, *F*(1.44,145.05) = 63.84, *p* < .001, η_p_^2^ = .39, 90% CI [.28, .47]; *M*_slope_ = .03, *SEM* = .004, *t*(104) = 10.05, *p* < .001, *d* = .98, 95% CI [.75, 1.21] and this effect was slightly stronger for fearful than for happy faces, *F*(1.59,160.68) = 5.64, *p* = .008, η_p_^2^ = .05, 90% CI [.01, .11]; fear *M*_slope_ = .05 ± .006, *t*(104) = 7.56, *p* < .001, *d* = .74, 95% CI [.52, .95]; happy *M*_slope_ = .03 ± .004, *t*(104) = 7.07, *p* < .001, *d* = .69, 95% CI [.48, .90]; *M*_slopediff_ = .015, *SEM* = .007, *t*(104) = 2.03, *p* = .045, *d* = .27, 95% CI [.006, .54]. Notably, there was only a trend for an interaction of Emotion Type with Age, *F*(1, 101) = 3.45, *p* = .07, η_p_^2^ = .03, 90% CI [0, .11] and no significant three-way interaction with Intensity, *F*(1.59,160.68) = 1.96, *p* = .15, η_p_^2^ = .02, 90% CI [0, .06]; see Figure S5.

### Self-Reported Mood Measures

There were no significant differences between age groups for measures on Trait Anxiety, *t*(102.4) = .97, *p* = .33, *d* = .19, 95% CI [–.19, .57]; State Anxiety, *t*(102.6) = .50, *p* = .61, *d* = .10, 95% CI [–.29, .48]; BDI, *t*(89.8) = −1.14, *p* = .26, *d* = −.22, 95% CI [–.61, 0.16]; short PANAS Positive, *t*(101.5) = −1.61, *p* = .11, *d* = −.31, 95% CI [–.70, 0.07]; or short PANAS Negative, *t*(96.9) = 1.73, *p* = .09, *d* = .34, 95% CI, [–.05, 0.72] questionnaires.

Trait Anxiety was correlated with a small number of measures in the emotion-WM task, but these effects were relatively weak and inconsistent when including versus excluding trials in which participants reported the wrong emotion type, which suggests no strong relationship between our behavioral measures and Trait Anxiety in the present sample.

## Discussion

We tested younger and older adults on novel precision emotion-WM and emotion-matching tasks and found age-related changes in the way emotional content was represented in WM. Specifically, older adults recalled fearful faces from WM as being less fearful than did younger adults, indicating an age-related attenuation in the representation of negative information in WM. Furthermore, older adults exhibited a positive interpretation bias whereby they were more likely to categorize low-intensity, fearful faces as being happy compared with younger adults. There were similarities between the patterns of results for the perceptual matching and WM tasks, but the results were relatively weak in the perception task and did not reach statistical significance. Separate from the changes in emotional bias, we found a general age-related impairment, such that older adults performed worse than younger adults in the WM and perceptual emotion-matching task for both happy and fearful faces.

By developing a novel task and analysis procedure, we revealed that the representation of emotional expressions in WM changes with age; older adults exhibited a systematic bias to remember fearful faces as less fearful than younger adults. There was no difference in bias for happy faces, suggesting that it is the representation of negative information in WM, and not positive information, that changes with age. Of interest, the pattern of results was dissociable from a general decline in WM accuracy: Older adults showed worse performance in WM and in the emotion-matching task for both happy and fearful faces.

The age-related difference in bias for fearful faces was partly driven by more ambiguous expressions closer to neutral emotion. Low-valence fearful faces were sometimes mistakenly interpreted as happy faces. Exclusion of such miscategorization trials dampened some of the relevant statistics, partly by lowering statistical power, but did not affect the overall pattern of results showing a shift toward a positivity bias in older participants. The interaction between Age and Emotion Type became a marginal trend, but the affective bias reflected in the shift in the psychometric curve remained robust after exclusion of incorrect responses. Furthermore, inspecting the curves suggests that the age-related bias occurred not only for low-intensity ambiguous faces, but also extended to faces displaying medium- and high-intensity fear. Overall, the findings suggest that the age-related difference in bias may partly reflect reinterpretation of ambiguous expressions, but is not confined to such a process, extending also to attenuating emotional content in stimuli with higher emotional valence.

To date, most studies that have explored age-related changes in emotional processing have used accuracy-based measures of bias, which give a measure of preferential processing (e.g., attending more to positive than negative stimuli), but leave open how the information was represented that lead to the behavioral effect. We note that, although performance impairments were greater for fearful than for happy faces, categorization and memory performance are often better for happy faces (e.g., Calder et al., 2003), suggesting effects related to perceptual features. Thus, our task was able to show how negative affective information in WM is attenuated with age, and that this was separate from age-related declines in WM.

Our task also enabled us to inspect age-related changes for interpreting ambiguous emotional expressions in WM. Older adults tended to judge low-intensity, ambiguously fearful faces as happier than did younger adults, suggestive of a positive interpretation bias in WM. Although participants were more likely to misinterpret low-intensity happy faces as fearful (cf. Phillips et al., 1998), older adults were more likely to report low-intensity fearful faces as happy, reflecting a tendency to interpret ambiguous expressions from WM positively. These results are consistent with emotion-categorization studies with ambiguous expressions (Bucks, Garner, Tarrant, Bradley, & Mogg, 2008; Kellough & Knight, 2012). Together, our findings indicate that older adults show an attenuation of negative information in WM, and a positive intepretation bias when dealing with ambiguous information. Our results are consistent with the positivity effect in aging (Carstensen, Isaacowitz, & Charles, 1999; Mather & Carstensen, 2005), but demonstrate that age-related differences can stem from multiple sources. With standard accuracy-based measures, it can be hard to determine why accuracy differences between positive and negative emotion conditions arise. New experimental paradigms and analysis methods designed to measure different types of emotional bias, such as those presented here, could lead to deeper insights into group and individual differences in affective processing.

Age-related differences in the perceptual matching task somewhat resembled the WM results, but did not reach statistical significance. Our perceptual matching task was primarily designed to ensure that older participants could perceive the task stimuli sufficiently well and were able to produce responses that reproduced emotional content with high levels of precision. The task worked well in this regard, showing high levels of accuracy. Of interest, however, though not statistically significant, the pattern of results is suggestive that positivity biases may even operate when making purely perceptual judgments. Unfortunately, because of the purpose for which we designed the perceptual matching task, the smaller number of trials may have precluded robust testing of this possibility. It will be interesting, therefore, for future studies to extend on the current findings to test for potential emotional biases in interpreting perceptual stimuli.

Three previous studies have reported that WM for emotional content is preserved in aging, regardless of the valence (Hartley et al., 2015; Mammarella et al., 2013; Truong & Yang, 2014), but the way they tested WM was fundamentally different from our task. Mammarella et al. (2013) tested WM for emotional and neutral words, whereas we used faces. Semantic meaning may be more similar across age groups, which might lead to a similar meaning-based memory benefit for emotional words (also see Truong & Yang, 2014). Hartley et al. (2015) used change-detection WM tasks with emotional faces, and found that older adults performed as well as younger adults in the emotional expression task but were impaired in the identity task. However, in the expression change-detection task, participants only had to recall the expressions without needing to remember visual features, which may have encouraged use of emotional expression labels. Furthermore, because they used an accuracy-based measure, it is unclear why there was a performance benefit. Another study using a judgment-based measure of performance found that older adults performed better on positive than negative images on a WM task, whereas younger adults showed the opposite pattern (Mikels et al., 2005). It should be noted that the task used in this study had participants judge whether the image encoded into WM was more or less emotionally intense than the subsequently presented “test” item (which were images of different things), and accuracy was based on concordance with emotional intensity ratings from an independent group of younger adults. Finally, Bermudez and Souza (2017) used a serial presentation WM task with positive, neutral, and negative images, and found an interaction between valence and age, revealing that older adults showed poorer performance on negative images than on positive and neutral images, consistent with the age-related positivity effect. In the current study, we showed that older adults had a deficit in both WM and emotion-matching tasks with a particular deficit for fearful faces, consistent with deficits in emotion recognition (Ruffman, Henry, Livingstone, & Phillips, 2008), and found age-related shifts in the affective content in WM unobtainable using accuracy measures alone.

Face stimuli in this study comprised images of young adults. Although a previous study found no own-age bias for recognizing emotional expressions in younger and older participant groups (Ebner & Johnson, 2009), it will be useful to extend the current findings using emotional faces of older adults. Another limitation of the current study was the focus on only fearful and happy emotional expressions. The precision-WM method we introduced should prove informative in charting to what extent biases are introduced in judging other emotional expressions, such as anger and disgust.

Although previous studies have reported a relationship between measures of anxiety and performance with emotional stimuli (e.g., see Yiend, 2010), we did not find any reliable results to suggest that this is the case for WM. It could be that we did not recruit participants with a large enough range of anxiety scores, or that our measures might correlate with depressed mood (for which we did not have a good range). It will be interesting for future researchers to test participants with a larger range of mood scores (e.g., patients) to test whether there are biases in WM for emotional material linked to mood, and if this changes with age. Another interesting possibility for future work is to test the specificity of our age-related performance deficits to emotional stimuli. It would be interesting to test participants on both the emotion-WM task and a comparable WM task with nonemotional features, such as faces morphed from male to female, to test if age-related deficits would be worse than or similar to WM for emotion-relevant features.

Our study employed a novel emotion-WM task that captured age-related impairments in cognition while revealing positive changes in emotional bias in WM that come with normal aging. Our findings provide support to the positivity-effect hypothesis in aging (Carstensen et al., 1999), revealing a more nuanced picture of the origin for this bias within WM. With our sensitive new approach, we were able to reveal multiple aspects of affective processing that undergo change in aging, including attenuation of negative information perception and a tendency for positive interpretation in WM. In future work, tasks and response methods that include continual measures of accuracy as well as measures of bias will be able to further reveal behavioral patterns in aging and characterize the emotional biases across individuals in mood and other psychological disorders.

## Supplementary Material

10.1037/emo0000481.supp

## Footnotes

1 To access the stimuli, please follow the instructions in the link, forward the email with permission to use the stimuli to the authors, and a link to download the images will be shared.
