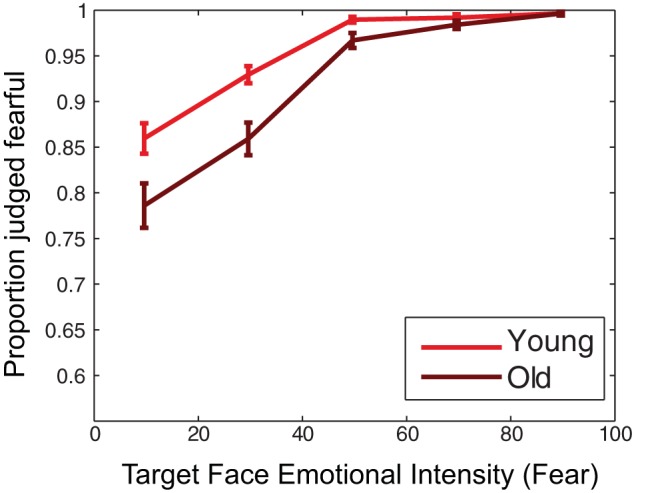# Correction to Mok et al. (2018)

**DOI:** 10.1037/emo0000604

**Published:** 2019-06-24

In the article, “Changing Interpretations of Emotional Expressions in Working Memory With Aging,” by Robert M. Mok, Jasper E. Hajonides van der Meulen, Emily A. Holmes, and Anna Christina Nobre (*Emotion*, 2018, advance online publication, http://dx.doi.org/10.1037/emo0000481), the plots for Figure 3a shifted incorrectly to the right. The error bars should be centered on 10, 30, 50, 70, and 90. The corrected figure appears below:[Fig fig1]

## Figures and Tables

**Figure 3 fig1:**