# Supplementary material for: Changing Interpretations of Emotional Expressions in Working Memory With Aging
Source: Emotion. 2018 Oct 15;19(6):1060–9. doi: 10.1037/emo0000481 (PMC6764502; doi:10.1037/emo0000481)
Supplement: Supplementary file 1 [file EmoAge_Emotion_sub_Supplementary_round3_final.docx]

**Supplementary Online Materials**

**Stimuli and Apparatus**

For each person (identity) in the stimulus set, one photograph was selected from each emotion type (fear, happy, neutral) in the mouth‑open configuration forming a set of 3 (see figure S1 and S2 for examples). Each photo was cropped using an elliptical mask (dimensions of rectangle: 506 x 650 pixels; dimensions of ellipse: 350 x 572 pixels). For cropping, images were loaded into Matlab and converted into grayscale. The elliptical mask was overlaid onto the photographs to create a black border and saved as images (see figure S1). These images were then loaded into Morpheus Photo Morpher. For each person in the face stimulus set, a set of faces morphed from neutral‑to‑fear and a set of images morphed from neutral‑to‑happy were produced. This resulted in 16 facial identities with two morphed emotion stimulus sets. Ten of the highest quality stimulus sets were selected for the main experiment (see figure S2 for examples of a set of high quality morphed happy and fearful expressions). The remaining six identities were selected for practice trials.

In the emotion WM task, faces were presented at the center of the screen and subtended 10° x 16.3°. In the emotion-matching task, faces were presented at the left and right side of the fixation cross on the horizontal meridian (centered at ~9.75° in lateral visual angle from fixation). In both tasks, the fixation cross was a plus sign (“+”) at the center of the screen subtending ~1.5°, and stimuli were presented on a black background.

Eye movements were monitored on‑line with an eye‑tracker (EyeLink 1000, SR Research, Ontario, Canada) recording at 500 Hz.

The task was programmed and run in Matlab v.7.10 (MathWorks) using the Psychophysics Toolbox v.3.0 package (Brainard, 1997). The task was presented on an LCD screen with a spatial resolution of 1680 by 1200 pixels and a refresh rate of 60 Hz, placed ~67.5 cm from the participant.

**Procedure**

Participants first completed five self‑report mood questionnaires on an iPad (Qualtrics online survey software; Qualtrics, Provo, UT), then proceeded to the main experimental task. The experimenter verbally explained each experimental task with a PowerPoint slide presentation, each of which was followed by practice trials. Participants were given one to two practice blocks of each task (10 trials per block). The practice tasks were the same as those in the main experiment, except that the facial identities were selected from the practice set. In the main experiment, all participants performed the emotional WM task followed by the emotional-expression matching task, with breaks in between.

**Trial Numbers**

After excluding trials with neutral (0% intensity) faces, there were 152 trials (76 per emotion-type condition) for the WM task, within which there were 16 trials per emotional intensity bin (12 trials for intensity bin 1, which excludes 0%). After removing misreported emotion trials, there were 72.8±.32 / 70.33±.60 (young/old) trials for the fearful face conditions and 67.1±.67 / 67.6±.67 (young/old) trials for the happy face conditions (out of 76 trials). Split into number of trials per emotional intensity bin, the mean number of trials per bin (young/old) were as follows: bin 1: 17.33±.37 / 17.45±.44, bin 2: 27.83±.34 / 26.37±.37, bin 3: 31.15±.15 / 30.67±.20, bin 4: 31.70±.09 / 31.57±.14, bin 5: 31.87±.05 / 31.86±.05 (note that bin 1 has 24 trials and others have 36 trials).

For the emotion-matching task, there were 38 trials (19 per emotion-type condition) after excluding trials with neutral faces, within which there were 4 trials per emotional intensity bin (3 trials for intensity bin 1, which did not include 0% emotional intensity). After removing misreported emotion trials, there were 18.30±.11 / 17.76±.21 (young/old) trials for the fearful face conditions and 18.48±.09 / 18.27±.12 (young/old) trials for the happy face conditions (out of 19 trials). Split into number of trials per emotional intensity bin, the mean number of trials per bin (young/old) were as follows: bin 1: 2.09±.14 / 1.86±.13, bin 2: 3.72±.07 / 3.20±.15, bin 3: 3.96±.03 / 4.00±.00, bin 4: 4.00±.00/ 4.00±.00, bin 5: 4.00±.00 / 3.98±.02 (note that bin 1 has 3 trials and the others have 4 trials).

**Maximum response time trials**

There were very few trials in which participants required the maximum response time (11s). Most participants did not have any of these trials, and the number of such trials was not significantly different between groups as reported below. Excluding these trials did not affect the results reported. The mean number of maximum response trials in the WM for fearful faces condition was *M*=.35, *SEM*=.15 (max=7) in the younger group and *M*=.51, *SEM*=.14 (max=4) in the older group (t(103)=-0.767, p=0.445, d=-0.15), and in WM for happy faces was *M*=.41, *SEM*=.12 (max=4) in the younger group and *M*=.33 , *SEM*=.13 (max=5) in the older group (t(99.3)=-1.43, p=0.15; d=-0.28). The mean number of maximum response trials in the emotion-matching task for fearful faces was *M*=.35, *SEM*=.08; (max=2) in the younger group and *M*=.53, *SEM*=.09 (max=3 trials) in the older group (t(100.4)=0.42, p=0.68, d=0.08), and for emotion-matching for the happy faces was *M*=.43, *SEM*=.10 (max=3) in the younger group and *M*=.45, *SEM*=.09; (max=3) in the older adults (t(103)=-0.19), p=0.85, d=-0.04).

**Supplementary figures**


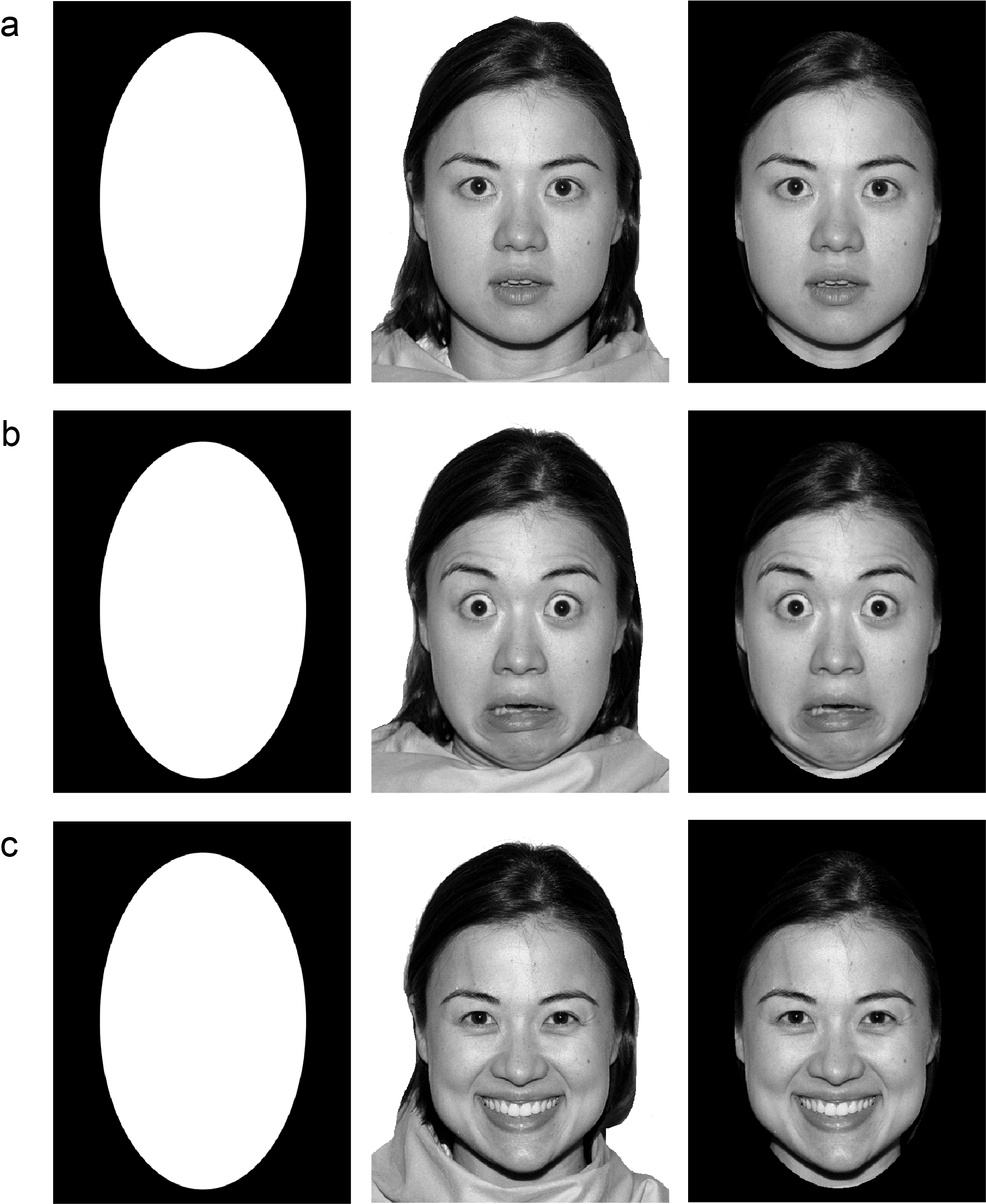


*Figure S1.* Cropping stimuli. An elliptical mask was laid over each photograph for (a) neutral (b) fearful and (c) happy faces. Faces presented are part of the NimStim stimulus set which are allowed for publication.


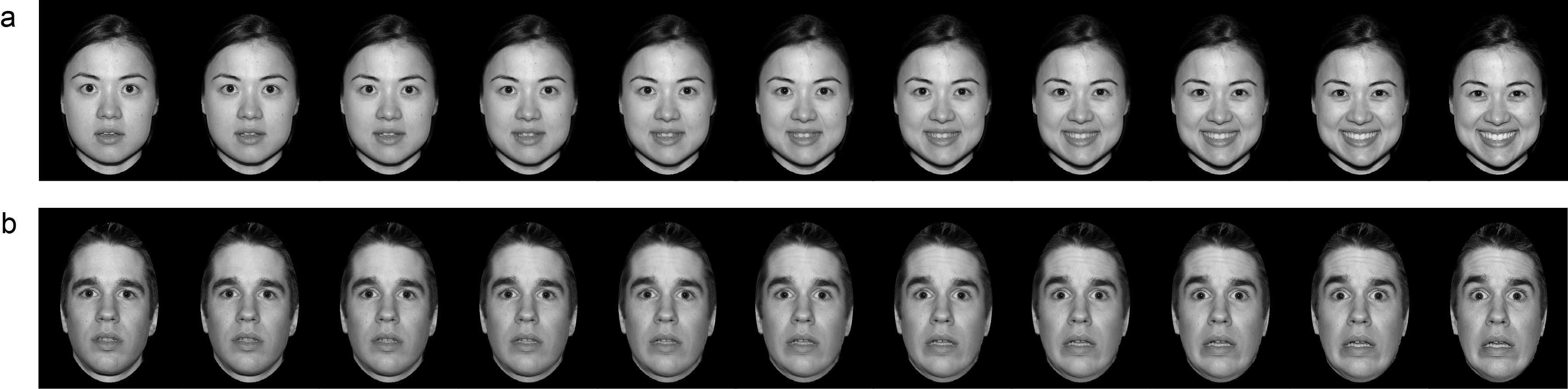


*Figure S2.* Examples of the full set of morphed images from 0 to 100% in 10% steps for a neutral‑to‑happy morph (a) and a neutral‑to‑fear morph (b). Faces are part of the NimStim stimulus set which are allowed for publication.

*
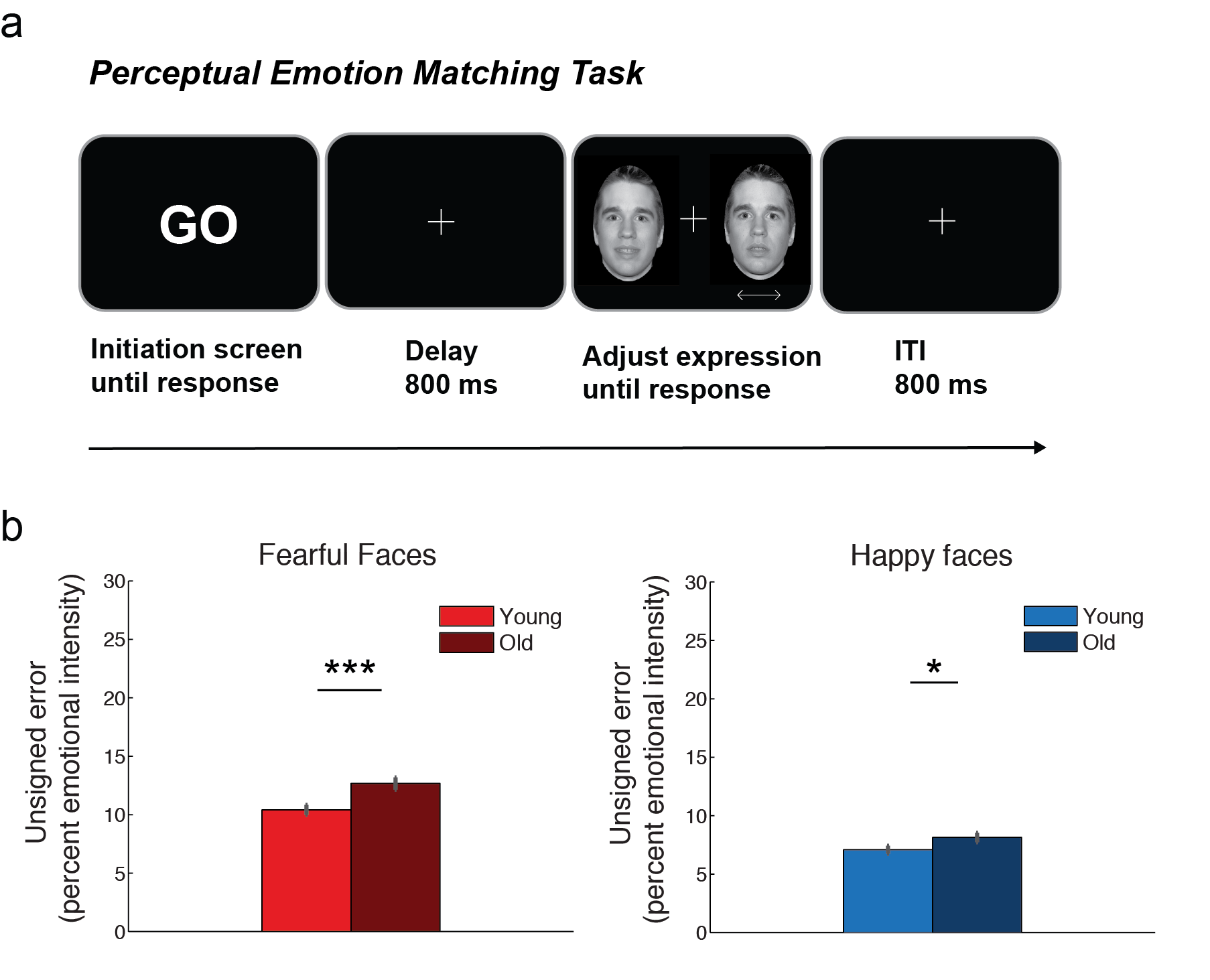
*

*Figure S3.* Emotion-matching task schematic and error results. In the perceptual emotion-matching task (a), participants initiated the trial with a mouse click, and were presented with an emotional face on the left and a neutral face on the right of the screen. Participants adjusted the emotional expression of the face on the right to match the emotion type and intensity of the face on the left using a trackball mouse. Emotion type was intermixed within blocks. Bar plots in (b) show emotion-matching error for fearful faces (left) in the young (light red) and old (dark red) groups and happy faces (right) in the young (light blue) and old (dark blue) participant groups. Error bars represent SEM *** p<.001, * p<.05. Faces presented are part of the NimStim stimulus set which are allowed for publication.

*
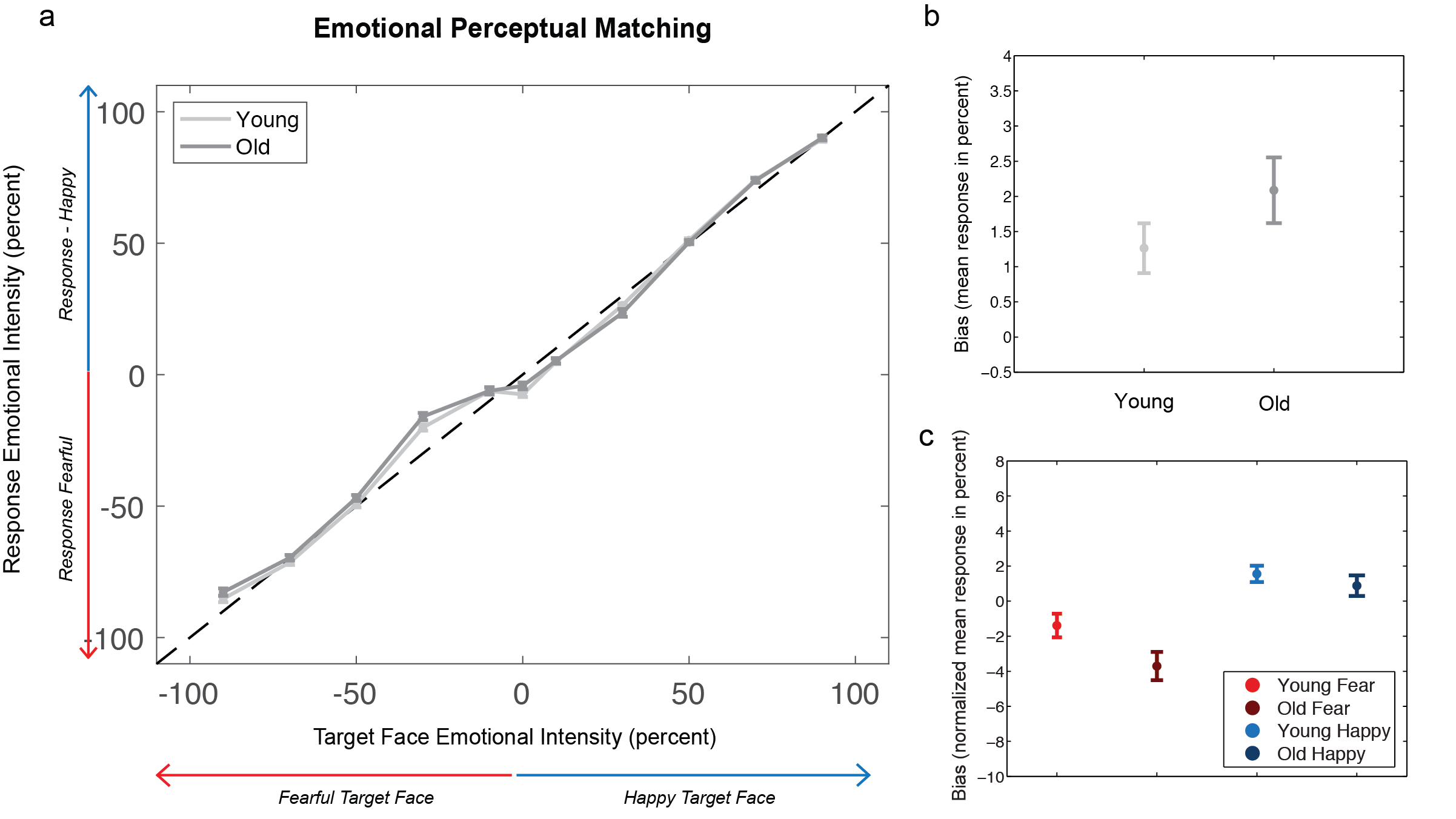
*

*Figure S4.* Emotional bias in perceptual-matching task. Participants’ responses are plotted as a function of the target face emotion type and emotional intensity in (a), with negative values representing intensity values of fearful faces and positive values representing intensity values of happy faces. Responses are binned into five equal bins for fearful faces, five bins for happy faces and a 0% bin with only neutral faces. The bias is shown in (b), computed by taking the mean of each participant’s raw psychometric curve. Bias for each of the emotion types separately in plot in (c). Conventions as in figure 2 in the main text.


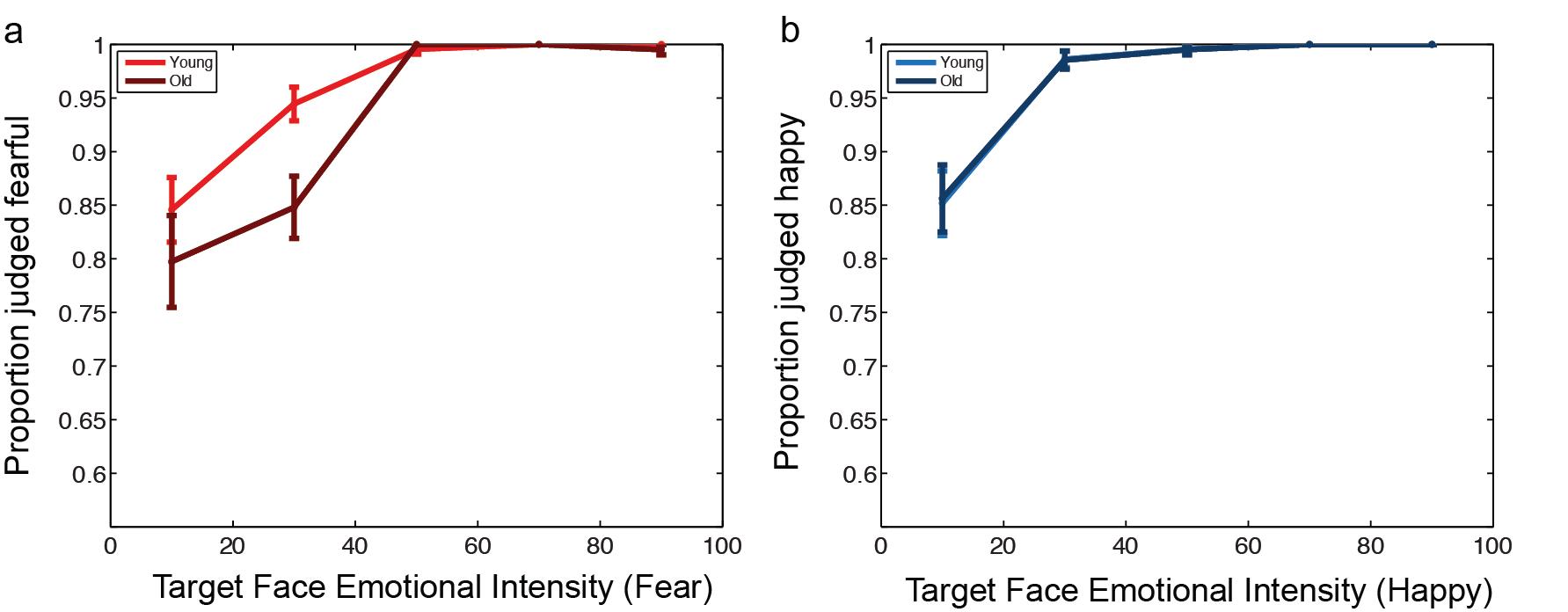


*Figure S5.* Proportion of trials correctly judged as fearful in the emotion-matching task are plotted for each emotional intensity bin from 1% to 80% in 20% steps in (a) and proportion of trials correctly judged as happy are plotted in (b) for younger and older participants.


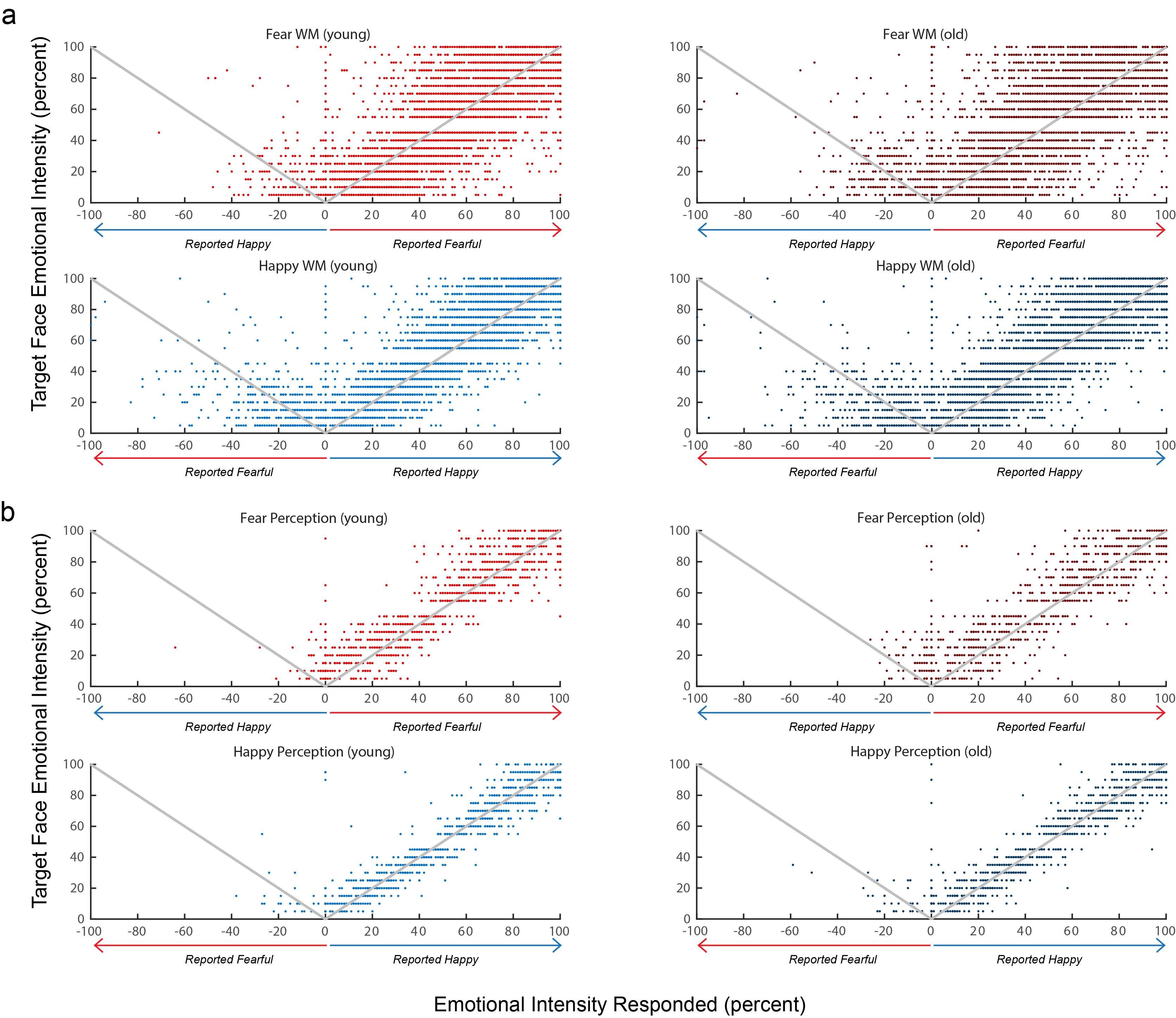


*Figure S6*. Scatterplots showing trials where participants correctly reported and misreported the emotion type for each Emotion and Intensity value condition. Scatter plots in (a) show target face emotional intensities plotted as a function of participant responses for emotional WM for fearful and happy faces, for young and older adults. Each point is an individual trial, and each scatter plot includes all trials in the specified condition in all participants within the age group presented. In all plots, the x-axis is the reported emotional intensity, y-axis is the target emotional intensity value; positive values correspond to the target emotion type intensities, negative values correspond to the other emotion type intensities. Responses to the correct emotion type lie to the right of zero, and responses to incorrect emotion type lie to the left of zero (note that the x-axes denoting the fearful face and happy face responses are flipped for the different emotion type conditions). Correct responses would lie on the identity line (y=x) on the right of each plot. A ‘mirrored’ version of the identity line (y=-x) is plot on the left side for reference (e.g. if participants interpreted a 20% happy face to be 20% fearful, it would lie on this line). Scatterplots in (b) show target face emotional intensities plotted as a function of participant responses for emotional-expression matching. Conventions as in (a).
